# Prevalence and correlates of depression, anxiety, and burnout among physicians and postgraduate medical trainees: a scoping review of recent literature

**DOI:** 10.3389/fpubh.2025.1537108

**Published:** 2025-07-08

**Authors:** Samuel Obeng Nkrumah, Medard Kofi Adu, Belinda Agyapong, Raquel da Luz Dias, Vincent Israel Opoku Agyapong

**Affiliations:** ^1^Department of Psychiatry, Dalhousie University, Halifax, NS, Canada; ^2^Department of Psychiatry, University of Alberta, Edmonton, AB, Canada

**Keywords:** physicians, residents, prevalence, burnout, depression, anxiety

## Abstract

**Background:**

The mental well-being of physicians is increasingly recognized as vital, both for their personal health and the quality of care they provide to patients. Physicians face a variety of mental health challenges, including depression, anxiety, and burnout, which have become prevalent issues globally. These mental health concerns are like those found in the general population but are particularly significant in the demanding healthcare setting.

**Objective:**

This review aims to explore the prevalence and correlates of depression, anxiety, and burnout among physicians and residents in training.

**Methods:**

A comprehensive literature review was conducted, searching databases such as Medline, PubMed, Scopus, CINAHL, and PsycINFO. The review focused on studies published from 2021 to 2024 that addressed the prevalence of these mental health conditions in physicians and residents. The findings, in line with the Preferred Reporting Items for Systematic Reviews and Meta-Analyses (PRISMA) guidelines, were summarized in detailed tables.

**Results:**

Following titles and abstracts screening, 196 publications were selected for full-text review, with 92 articles ultimately included in the analysis. The results revealed significant variability in the prevalence of burnout, depression, and anxiety. Burnout rates among physicians ranged from 4.7 to 90.1% and from 18.3 to 94% among residents. Depression prevalence ranged from 4.8 to 66.5% in physicians and from 7.7 to 93% in residents. Anxiety rates were between 8 and 78.9% in physicians and 10 to 63.9% in residents. Notably, women reported higher rates of all three conditions compared to men. Key factors influencing these mental health conditions included demographics (age, gender, education, financial status, family situation, occupation), psychological conditions, social factors (stigma, family life), work organization (workload, work conditions), and COVID-19-related issues (caring for COVID-19 patients, fear of infection, working in high-risk areas, concerns about personal protective equipment (PPE), and testing positive).

**Conclusion:**

This review indicates a high prevalence of burnout, depression, and anxiety among physicians and residents, with female participants consistently showing higher rates than males. These findings can guide policymakers and healthcare administrators in designing targeted programs and interventions to help reduce these mental health issues in these groups.

## 1 Introduction

In the demanding and high-stress environment of the healthcare profession, the mental well-being of physicians is increasingly recognized as a critical component of both individual health and patient care quality. Ensuring a robust and capable cadre of physicians is fundamental to the effectiveness of any nation’s healthcare infrastructure (1). The suboptimal mental health and wellness of healthcare personnel have organizational repercussions for patient safety, satisfaction, and overall experience (1). Physicians can experience a wide array of mental health conditions alongside various challenges impacting their overall wellness, including burnout. Like the broader populace, prevalent mental health issues among physicians include depression, anxiety, and burnout (2). Depression, anxiety, and burnout represent significant challenges faced by physicians worldwide (3–9), impacting not only their personal lives but also their professional performance and the broader healthcare system. As frontline providers, physicians bear the crucial duty of delivering top-tier medical care to patients amidst the intricacies of healthcare systems, rapidly evolving medical knowledge, and the emotional rigors of patient engagements. Intense work settings, substantial workloads, extended shifts, resource constraints, organizational changes, and a culture characterized by blame and apprehension have all been identified as contributing elements (10–12), increasing the susceptibility of physicians to mental health issues. Physicians and residents often avoid seeking mental health support due to stigma and concerns over their professional reputations. Many fear that acknowledging psychological issues such as burnout, anxiety, or depression might be seen as a weakness (2) and negatively impact their careers. A 2018 study by Aaronson et al. identified key barriers to mental health care access during residency, highlighting a lack of time, confidentiality concerns, and potential career consequences as major deterrents (13). Additionally, notable medical professionals have publicly discussed their own mental health struggles, further illustrating the damaging effects of stigma within the medical field (2).

Depression ranks as the primary contributor to global ill health and disability. It is characterized by persistent feelings of sadness, fatigue, hopelessness, loss of appetite, and loss of interest or pleasure in activities (14), and it is a prevalent mental health condition among physicians. Approximately 280 million people in the world have depression, and more than 700, 000 people die due to suicide every year (14). Studies consistently report higher rates of depression among physicians compared to the general population (15–17). Numerous people grappling with mental health difficulties encounter insufficient support structures and are discouraged by the social stigma attached to such issues, impeding their capacity to access the essential treatment needed to pursue fulfilling and productive lives, a circumstance in which physicians are not exempt. Mata et al. conducted a significant systematic review and meta-analysis that brought together findings from 54 distinct cross-sectional and longitudinal studies encompassing more than 17,500 resident physicians across 18 nations. Regardless of the country or specialty under investigation, similar rates of depression symptoms among physicians were observed. The combined assessment of depression caseness, indicating the proportion of physicians exhibiting clinically significant depressive symptoms, was calculated at 28.8% (with a 95% confidence interval of 25.3–32.5%) (18).

Anxiety is another common mental health challenge faced by physicians. In 2019, approximately 301 million individuals globally experienced anxiety disorders, which stood as the most prevalent among all mental health conditions, characterized by feelings of apprehension, worry, and tension (19). The pressure to make critical decisions, maintain clinical competence, and provide optimal care in high-stakes situations can contribute to heightened anxiety levels among physicians. Moreover, the rapid pace of medical advancements, coupled with the need to keep abreast of new diagnostic and treatment modalities, can exacerbate feelings of uncertainty and insecurity, further impacting physician well-being. Numerous studies have demonstrated a higher prevalence of anxiety among physicians (6, 20, 21). A cross-sectional study conducted by Gong et al., among 2,641 physicians working in public hospitals in China found that approximately 25.67% of doctors displayed signs of anxiety, while 28.13% exhibited symptoms of depression, and 19.01% experienced both anxiety and depression. These mental health challenges among the surveyed physicians were linked to self-reported declines in physical well-being, instances of workplace violence, extended work hours surpassing 60 per week, frequent night shifts occurring twice or more weekly, and a lack of consistent physical activity (22).

Freudenberger, a psychologist, introduced the notion of burnout in a paper titled “Staff Burnout,” (23) and its recognition gained traction with the introduction of the Maslach Burnout Inventory (MBI) assessment tool by Maslach and Jackson in 1981 (24). Burnout arises as an adverse workplace condition due to prolonged exposure to stress associated with one’s job (25). It is often described as a syndrome of emotional exhaustion, depersonalization, and reduced personal accomplishment (26). It is particularly prevalent among individuals who are employed in roles that involve frequent direct interaction with others (27) and is prevalent among physicians across various specialties and practice settings (28–33). The chronic stressors inherent in medical practice, such as heavy workloads, time pressures, and the emotional toll of patient care, can lead to feelings of burnout over time. Physician burnout has garnered more focus over the years (34, 35). Physicians experiencing burnout tend to make more medical errors (36, 37), are more inclined to leave their positions (38), express lower job satisfaction (39), and have implications on healthcare costs (37). Also, burnout among physicians has been associated with poorer patient perceptions of care (40), making it a significant concern for healthcare organizations and policymakers. In a cross-sectional study conducted by O’Dea et al., among 683 general practitioners (constituting 27.3% of practicing Irish general practitioners), 52.7% reported significant emotional exhaustion, 31.6% scored high on depersonalization, and 16.3% exhibited low levels of personal accomplishment. Overall, 6.6% experienced all three symptoms, meeting the criteria for burnout (41).

Despite growing recognition of the importance of addressing mental health issues among physicians, there remains a need for a comprehensive understanding of the prevalence and correlates of depression, anxiety, and burnout within this population. The primary objective of this scoping review is to map the existing literature on the prevalence and correlates of depression, anxiety, and burnout among physicians. It aims to provide insights into the scope and magnitude of mental health challenges faced by physicians and medical trainees (residents and fellows) across different specialties, practice settings, and geographic regions. Specifically, this scoping review will address the following: The prevalence of depression, anxiety, and burnout among physicians across different specialties and practice settings; Influence of associated factors, e.g., demographic characteristics (e.g., age, gender), professional factors (e.g., years of experience, work hours), social and psychological factors on the prevalence of depression, anxiety, and burnout among physicians. The review seeks to offer critical insights for healthcare policymakers, administrators, educators, and researchers. The findings can serve as a foundation for developing targeted interventions and support systems aimed at improving the mental health and well-being of physicians and residents. In turn, this not only enhances patient care but also boosts the overall efficiency and effectiveness of the healthcare system.

## 2 Methods

### 2.1 Search strategy

A literature search was conducted to look for articles that explored the prevalence and correlates of depression, anxiety, and burnout among physicians, residents, and fellows. The databases Medline, PubMed, Scopus, CINAHL, and PsycINFO were searched in the second week of April 2024, focusing on studies published from January 1, 2021, to May 1, 2024. Only articles written in English were considered. The search aimed to find studies examining the prevalence of each condition separately (e.g., just depression) and those addressing two or all three conditions together (e.g., depression, anxiety, and burnout). The search terms included: “prevalence of depression,” “prevalence of anxiety,” “prevalence of burnout,” “depression,” “burnout,” “anxiety,” “prevalence,” “physicians,” “doctors,” “medical practitioners,” and “resident physicians.” Appendix 1 provides some examples of the search strategy. Two reviewers (S.O.N and M.A) independently searched the databases and reviewed the articles. The screening process had two stages: an initial screening of titles and abstracts to assess relevance, followed by a full-text screening. Articles meeting the initial inclusion criteria advanced to the full-text screening phase. Disagreements were resolved by consulting a third reviewer (B.A). The review followed the Preferred Reporting Items for Systematic Reviews and Meta-Analyses (PRISMA) guidelines (42).

### 2.2 Inclusion and exclusion criteria

Articles were included based on the following criteria: (1) studies published between 2021 and 2024 in English, (2) a clearly defined sample of physicians, residents, and fellows, (3) clear reporting of prevalence for depression, burnout, or anxiety, and (4) a clearly stated study design, such as cross-sectional, cohort, mixed-method, transverse, or longitudinal. Studies that did not sample physicians, were reviews, protocols, or experimental studies were excluded. The study measures of interest were depression, anxiety, or burnout. Thus, studies were excluded if they did not report prevalence or did not focus on physicians or residents. Excluded populations were: (1) Medical students (2) individuals from other health-related fields (e.g., dentistry, pharmacy, nursing, allied health sciences), and (3) other healthcare professionals.

### 2.3 Data extraction process

Information was extracted and summarized in a table, which included the author’s name, publication year, country of study, study population, sample size, response rate, tools used to assess mental health conditions (depression, anxiety, or burnout), prevalence of these conditions, and any associated factors (Table 1).

**Table 1:** Prevalence and correlates of burnout among physicians and postgraduate medical trainees in studies conducted from 2021 to 2024.

Author’s name	Year of publication	Country study was conducted	Study population	Sample size	Response rate	Tool for measuring burnout	Prevalence of burnout	Associated factors
Appiani et al. (99)	2021	Argentina	Physicians	440	68.63%	MBI	Overall prevalence: 73.5%	Increasing burnout: having less seniority being a resident caring for patients with potential or confirmed COVID-19 infection Having transient COVID-19-like symptoms working 24-h shifts
Fiabane et al. (51)	2023	Italy	Physicians	18,516	6.5%	CBI	Overall prevalence: 18.5%	Increasing burnout: female sex younger age shorter job tenure trainee status higher PHQ-8 and GAD-7 scores
Matsuo et al. (52)	2021	Japan	Residents	4,754	12.7%	MBI-GS	Overall prevalence: 28%	Increasing burnout: excessive working hours low autonomy communication problems at the workplace complaints from patients peer competition anxiety about the future
Hain et al. (100)	2021	South Africa	Doctors	213	45%	MBI	Overall prevalence: 65.8%	Increasing burnout: Female gender Occupational rank planning to leave the public sector in the next 2 years.
Crudden et al. (101)	2023	Ireland	Physicians	2,160	21.9%	MBI	Overall prevalence: 42%	Increasing burnout: elevated symptoms of depression on the DASS depressive symptom subscale (EE) higher rates of face-to-face patient contact (EE) Decreasing burnout: rofessional efficacy
Ofei-Dodoo et al. (53)	2021	USA	Physicians	113	45.6%	MBI	Overall prevalence: 50.4%	Increasing Burnout: personally, treating patients suspected or confirmed to have COVID-19.
Al-Humadi et al. (54)	2021	USA	Physicians and residents/fellows	1,379	16.3%	MBI (2 single items)	Overall prevalence: 19.6%	Increasing burnout: History of depression or anxiety younger age female gender (physician) higher number of on call time
de Mélo Silva Júnior et al. (102)	2022	Brazil	Residents	1,989	71.4%	MBI 2-items version	Overall prevalence: 37%	Associated with increasing burnout: lower age and leisure time male sex longer duty hours absence of day off provision of care without supervision choice of the wrong specialty poor learning sychological abuse depression
Rubin et al. (55)	2021	Canada	Physicians	151	84.1%	WBI	Overall prevalence: 65.4%	Increasing burnout: perception of inadequate staffing levels being treated unfairly in the workplace.
Che et al. (56)	2023	China	Physicians (anesthesiologists)	8,850	74.93%	MBI-HSS	Overall prevalence: 52.7%	Increasing burnout: redeployment outside normal professional boundaries^*^ depression anxiety PTSD Protective factors: resilience good institutional support
Alwashmi et al. (103)	2021	Saudi Arabia	Physicians(psychiatrist)	101	Not reported	MBI-HSS	Overall prevalence: 80.2%	Significantly increasing burnout: gender (female) -working in tertiary centers psychiatrist in-training (junior and senior residents)
Kuriyama et al. (104)	2022	Japan	Physicians	1,173	18.2%	Mini-Z	Overall prevalence: 31.8%	Increasing burnout: having no partner^*^ shortage of PPE*
Carneiro Monteiro et al. (105)	2021	Brazil	Psychiatry residents	185	62%	MBI-HSS	EE:60% DP:54.8% PA:33%	Significantly associations: nature of relations to the institutions (EE) nature of relationships with preceptors/supervisors (EE, DP) quality of relationship with family (EE, DP) age (DP)
Jiménez-Labaig et al. (57)	2021	Spain	Residents and Specialists	243	26.6%	MBI-HSS MP	Overall prevalence: 25.1%	Increasing burnout: Younger age^*^ perceived lack of leisure time or vacation time^*^ poor perception of work life balance*
Steil et al. (106)	2022	Brazil	Residents	3,071	10%	OLBI	Overall prevalence: 48.6%	Associated with burnout: avoidance of seeing patients with confirmed or suspected cases of COVID-19 lack of supervisor support for the treatment of COVID-19 patients working in a wing with high risk of contamination belief that PPE is not efficacious fear of getting COVID-19 and transmitting it to significant others having personal relationships impaired since the pandemic
de Mélo Silva Júnior et al. (107)	2023	Brazil	Physician residents	First cohort(pre-COVID): 524 Second cohort (pandemic group): 419	Not reported	2-item MBI	Overall prevalence: pre-COVID cohort: 37% pandemic cohort: 26.1%	No information provided
Pogosova et al. (108)	2021	Russia	Physicians	108	Not reported	MBI-HSS	EE: high-50%, moderate-33% DP:34.1% reduced PA:37.5%	Increasing burnout: being female (EE)
Rahimaldeen et al. (109)	2021	Saudi Arabia	Physicians(pediatricians)	386	65%	CBI	Overall prevalence: 80.5%	Increasing burnout: female gender being junior pediatrician being younger pediatricians
Tipwong et al. (59)	2024	Thailand	Physicians	227	Not reported	PFI	Overall prevalence: 30.7%	Negatively predicting burnout: clinical teaching self-efficacy
Hamdan et al. (60)	2023	Jordan	Residents and specialist surgeons	180	75%	aMBI	Overall prevalence: 45.2%	Associated with burnout: -age positively correlated with PA and negatively with EE and DP number of children negatively correlated with DP years of experience among specialists negatively correlated with EE and DP
Youssef et al. (61)	2022	Lebanon	Physicians	398	Not reported	CBI-Arabic version	Overall prevalence (high and moderate levels): 90.1% PB:80.4% WB:75.6% CB:69.6%	Increasing burnout: female gender younger age being single having a dependent child, living with older adult or a family member with comorbidities insufficient sleeping hours working in a public health facility limited years of professional experience lack of previous experience in a pandemic extensive working hours Decreasing burnout: being married financial well-being good health history of COVID-19 previous pandemic experience
Nonaka et al. (62)	2022	Japan	Physicians	First survey: 1,251 Second survey: 1,241	First survey: 22.6% Second survey: 25.9%	Single-item Mini-Z	Overall prevalence: -First survey: 34.6% -Second survey: 34.5%	Increasing burnout: history of self-quarantine^*^ Not associated with exacerbation: being a woman^*^ being a clinical resident^*^ having worked in a prefecture under a state of emergency^*^
Turalde et al. (63)	2022	Philippines	Residents	120	71.67%	MBI	Overall prevalence: 94% EE:34.8% DP:8.14% Low PA:93%	Associated with burnout: the lack of compensation (EE) number of on-duty days (EE, DP)
Singh et al. (64)	2022	Canada	Physicians	634	44%	MBI	Overall prevalence: 72.9% EE:64.9% DP:47.2% Low PA:27.2%	Increasing burnout: working in a hectic or chaotic atmosphere feeling unappreciated on the job reporting poor or marginal control over workload not being comfortable talking to peers about workplace stress decreasing burnout: older age
Alrawashdeh et al. (48)	2021	Jordan	Physicians	973	Not reported	BMS	Overall prevalence: 57.7%	Increasing burnout: female gender working at highly loaded hospitals working for long hours doing night shifts lack of sufficient access to PPE being positively tested for SARS-CoV-2.
Blazin et al. (47)	2021	USA	Physicians	132	40%	MBI	Overall prevalence: 28%	Increasing burnout: frequent meetings insufficient support staff workflow interruptions
Wang et al. (65)	2021	China	Physicians	1813	90.7%	MBI	Overall prevalence: 82.1%; severe burnout: 38.8%	Increasing burnout: difficulty in making treatment decisions Protective factors: higher number of children higher “income satisfaction”
Carlson et al. (98)	2021	USA	Physicians	186	56%	2-item MBI	Overall prevalence: 26%	Positive association with burnout: hours worked in a typical week
Medina-Ortiz et al. (91)	2022	Venezuela	Physicians	150	Not reported	MBI	Overall prevalence: 76.7%	Increasing burnout: higher number of years working in the hospital lower job satisfaction
Nimer et al. (66)	2021	Jordan	Residents	481	Not reported	CBI	Overall prevalence: 77.5%; severe burnout: 16.2%; moderate burnout: 61.3%	Increasing burnout: psychological stress longer working being obstetrics/gynecology residents
Celik et al. (67)	2021	Turkey	Physicians (surgeons)	3,815	16.1%	MBI	Overall prevalence: 69.1%; severe burnout: 22.0%	Factors independently associated with Burnout: working in a training and research hospital or state hospital^*^ working ≥ 60 h per week^*^ less frequent participation in social activities*
Sharp et al. (97)	2021	USA	Fellows	976	51%	MBI two-item measure	Overall prevalence: 32%	Increasing burnout: Working more than 70 h in an average clinical week burdens of electronic health record (EHR) Documentation Decreasing burnout: access to mental health services coverage system in the case of personal illness or emergency
Nguyen et al. (92)	2022	USA	Physicians	400	13%	MBI-HSS	Overall prevalence: 57%	Increasing burnout: increased feelings of burnout due to the COVID-19 pandemic (EE, PA) ^*^ total hours of work per week (EE, PA) * younger age (EE, PA) *
Bean et al. (93)	^2022^	USA	Residents	1,298	22.8%	2-item MBI	Overall prevalence: 35.8%	Increasing burnout: Residents’ perception of not having adequate time for personal/family life Residents who reported inappropriate clerical burden working more than 50 h/wk. on inpatient rotations Protective against burnout: Faculty support performing activities that led residents to choose physical medicine and rehabilitation as a specialty
Hagqvist et al. (44)	2022	Sweden	Physicians	6,699	41%	BAT	Overall prevalence: 4.7%	Increasing burnout: working in the emergency department junior physicians
Boland et al. (68)	2023	UK and Ireland	Physicians	815	66.8%	MBI-HSS (MP)	Overall prevalence: 39.2%	Increasing burnout: formal supervision in palliative medicine high levels of depressive symptoms working over 40 h per week high-risk alcohol consumption Decreasing burnout: staff grade or trainee status higher perceived level of support
Kondrich et al. (95)	2022	USA and Canada	Physicians	416	49.5%	MBI	EE: 34.9% DP: 33.9% PA: 20%	Associated with burnout: lack of appreciation from patients^*^ lack of appreciation from supervisors^*^ perception of an unfair clinical work schedule^*^ issatisfaction with promotion opportunities^*^ feeling that the electronic medical record detracts from patient care^*^ working in a non-academic setting^*^
McGarry et al. (94)	2024	USA	Physicians	386	21.6%	CBI	Overall prevalence: 55.4%	Associated with positive burnout: inadequate compensation inadequate opportunity to process trauma
Doe et al. (69)	2024	USA	Residents	11,570	Not reported	MBI	Overall prevalence: 36.4%	Increasing burnout: female gender white race educational debt exceeding $250,000 Decreasing burnout: -being black and Asian race smaller program size
Keith (70)	2023	Canada	Physicians	847	50%	MBI-HSS (MP)	Overall prevalence: 58.9%	Increasing burnout: female pathologists (Significantly higher EE and lower PA)
Chan et al. (45)	2021	Canada	Physicians (Urologists)	609	17.2%	MBI	Overall prevalence: 31.8% EE:8.0% DP:31.8% low PA:10.6%	Increasing burnout: urologists under financial strain female urologists early-to-mid-career urologists.
Alenezi et al. (71)	2022	Saudi Arabia	Residents	426	77.45%	MBI-HSS	Overall prevalence (high on all subscales): 18.31% High EE: 57.51% High DP: 36.62% High PA: 12.91% moderate EE: 28.87% moderate DP: 32.63% moderate PA: 33.57% High on at least one subscale of burnout: 81.22%	Increasing burnout: lack of physical exercise (EE, DP, PA) having less than 3 weekends on-call per month (EE) dissatisfaction with work–life balance (EE, DP) time pressures and deadlines (EE) work overload (EE) inability to participate in decision-making (EE) inability to make full use of their skills and abilities (EE, PA) work centered life (EE) difficulty in maintaining relationship with their superiors (EE, DP)
Kurzthaler et al. (72)	2021	Austria	Physicians (GP vs. OS)	481 (252 GP and 229 OS)	Not reported	CBI	Overall prevalence (GP vs. OS): Intermediate:43.8% vs. 39.8% High: 26.9% vs. 22.0%	Predictors of burnout: being single financial problems experienced during COVID-19 stigmatization because of treatment of SARS-CoV-2-positive patients facing violence in patient care longer working hours during the pandemic.
Marques-Pinto et al. (73)	2021	Portugal	Physicians	43,983	9,176 (29%)	MBI	EE:66% DP:33% decrease-PA:39%	Predictors of burnout: organizational resources (EE, DP)^*^ demands of the relationship with the patients (EE, DP)^*^ work schedule (EE, DP)^*^
Yuan et al. (74)	2023	Canada	Resident physicians	345	48%	MBI-HSS	Overall prevalence: 58%	Decreasing burnout: having dependent being IMG being racial minority
Mcloughlin et al. (75)	2022	Ireland	Residents (psychiatry trainees)	510	21%	aMBI	Overall prevalence: 65%	Associated with burnout: staff shortages longer hours less experience.
Werdecker et al. (76)	2021	Germany	Physicians (GP)	548	Not reported	CBI	PB:35.2% WB:26.6% P_a_B:12%	Increasing burnout: being female (PB) working as an employed physician (PB). working in a single practice (PB, WB, PaB)
Shalaby et al. (77)	2023	Canada	Resident Doctors	1,594	9.8%	MBI	Overall prevalence: 58.2%	Associated with burnout: working more than 80 h/week (high EE and ID) being dissatisfied or being neither satisfied nor dissatisfied with a career in medicine (high EE and DP) agreeing that the residency program has enough strategies aimed at resident well-being in place (EE, ID) young age of residents (low PF)
Salihu et al. (78)	2023	Nigeria	Resident doctors	185	90.1%	MBI-HSS MP	High EE: 21.6% High DP: 13.6% Low PA: 30.7%	Association with burnout: Being a younger resident doctor aged 31–35 (EE, DP) duty hours >50 h per week (DP) presence of work-related stress (DP)
Rashid et al. (79)	2022	Bangladesh	Doctors	185	90.81%	MBI-HSS	overall prevalence: 55.4% High EE: 95.8% High DP: 98.2% Reduced PA: 97%	Increasing burnout (high levels in all 3 domains EE, DP, PA): Younger age (25–29 years) being female working as a medical officer
Gajjar et al. (80)	2022	Canada	Physicians	First survey (March 2020): 1,400 Second survey (March 2021): 2,638	First survey: 76.3% Second survey: 75.9%	Validated, single-item, self-defined burnout measure (1-no symptoms of burnout to 5-completely burned out).	Overall prevalence: -First survey:28% -Second survey: 34.7%	Increasing burnout: -patient expectations/patient accountability reporting and administrative obligations practice environment as the three factors that contributed most to burnout.
Ghazwani (81)	2022	Saudi Arabia	Physicians	51	86%	MBI-22 point scale	Overall prevalence: <25% EE: 18.2% DP: 25% Reduced PA: 25%	Increasing burnout (in all 3 domains EE, DP, PA): having less (<5 years) experience attending more patients (5–10/day) on all the three domains of burnout.
Shahi et al. (82)	2022	Nepal	Resident Doctors	410	84.6%	MBI	Overall prevalence: 42.4% High EE: 16.6% High DP: 15.9% Reduced PA: 9.8%	Independently increasing burnout: Gender (male) marital status having children specialty year of residency specialties hours of work per week (≥80 h)
Pawłowicz-Szlarska et al. (83)	2022	Poland	Physicians	225	43%	aMBI	High EE: 39.2% High DP: 38.1% Reduced PA: 21.6% Medium level in all 3 dimensions: 26.8% High levels in all 3 dimensions: 8.2%	Increasing burnout: excessive bureaucracy in healthcare systems rush at work overtime work
Fumis et al. (84)	2022	Brazil	Physicians	62	82%	MBI	Overall prevalence: 37.2% High EE: 51.0% High DP: 51.0% Reduced PA: 96.1%	No information provided
Ghoraishian et al. (85)	2022	Iran	Physicians (Surgeon) and Residents	180	Not reported	MBI	Overall prevalence: 50.0%	Significant associations with burnout: younger age lower academic rank or being a resident working in the public sector spending less time in leisure and sports activities.
Passos et al. (86)	2022	Brazil	Residents	139	49.26%	MBI	Overall prevalence: 73.1% EE: 44.8% DP: 64.2% PA: 47.8%	No association between overall burnout level and all analyzed variables -current year in the residency program (EE) the use of antidepressant/hypnotic medication (EE) current work routine (DP) having children (PA)
Kwan et al. (96)	2021	Hong Kong	Doctor/residents	2,879	Doctors:284 (9.9%) Residents-in-training: not reported	CBI	PB:72.6% WB:70.6% CB:55.5%	Increasing PB: engagement in longer working hour(s) per week working in Hospital Authority clinics Decreasing PB: Older age possession of a first university degree in medicine possession of Academy fellowship status Increasing WB: Being single, separated, or divorced longer working hour(s) per week
Seda-Gombau et al. (50)	2021	Spain	Physicians	150	27%	MBI for medical professionals	Time1: Overall prevalence:7.5% EE:37.5% DP:32.5% PA:27.5% Time 2: Overall prevalence: 10% EE:55% DP:30% PA:27.5% Time 3: Overall prevalence:50% EE:77.5% DP:70% PA:67.5%	Increasing burnout: Age (being older) Having children
Doolittle et al. (46)	2021	USA	Physicians	1,021	33%	ProQol	Overall prevalence: 52%	Increasing burnout: being a woman single physicians Decreasing burnout: older age Exercise (3 times per week for 20 min)
Khan et al. (87)	2024	South Africa	Doctors	430	68%	OLBI	Overall prevalence: 78%	Significant association with burnout: being a medical intern or community-service medical officer being in the lowest income band using alcohol to manage work-related stress experiencing high conflict at work high role ambiguity and role conflict
Sobczuk et al. (88)	2024	Poland	Physicians	228	Not reported	MBI-HSS	Overall prevalence: 74.9% EE: 64.5% DP: 37.0% PA: 43.1%	Increasing burnout: bureaucracy and administrative duties overload admissions of many patients poor work culture night/on-call duties
Pius et al. (89)	2023	Nigeria	Doctors	685	38.1%	CBI	PB:62.2% WB:52.2% P_a_B:27.5%	Increasing burnout: female gender less than 6 years of work experience working for at least 71 h in a week
Baptista et al. (58)	2021	Portugal	Physicians	225	Not reported	CBI	PB: 65.9% WB:68.7% P_a_B: 54.7%	Increasing burnout: higher levels of depression (PB, WB, PaB)^*^ higher anxiety levels (PB, WB)^*^ being female (PaB)^*^ having worked for 6 to15 years (PaB)^*^ reduction in monthly income inversely correlated with PaB^*^
Oluwadiya et al. (90)	2023	Nigeria	Physicians	256	60.5%	MBI-ES	Overall prevalence: 57.7%	Associated with burnout: religion (Muslims) (EE) geopolitical zone of practice (working in the north)-(EE) enjoyment of academic writing (EE) apathy toward teaching (EE) university ownership number of published peer-reviewed articles (EE) salary, and supplementary income (EE) number of weeks spent teaching in a year (DP, PA) teaching hours/week (DP, PA)

MBI, Maslach Burnout Inventory; MBI-HSS, Maslach Burnout Inventory-Human Services Survey; MBI-GS, Maslach Burnout Inventory-General Survey; MBI-ES, Maslach Burnout Inventory for Educators; aMBI, abbreviated-Maslach Burnout Inventory; CBI, Copenhagen Burnout Inventory; OLBI, Oldenburg Burnout Inventory; ProQol, Professional Quality of Life Scale; BMS, 10-Item Burnout Measure-Short version; BAT, Burnout Assessment Tool; Mini-Z, Mini-Z Burnout Assessment; WBI, Well-Being Index; PFI, Professional Fulfillment Index; PB, Personal-related Burnout; WB, work-related burnout; PaB, patient-related burnout; CB, client-related burnout; EE, emotional exhaustion; DP, depersonalization; PA, personal accomplishments; ID, interpersonal disengagement; PF, professional fulfillment; GP, General Practitioner; MP, Medical Personnel; OS, Other Specialties; PPE, Personal Protective Equipment.

* Multivariable analysis.

## 3 Results

**Figure 1:**
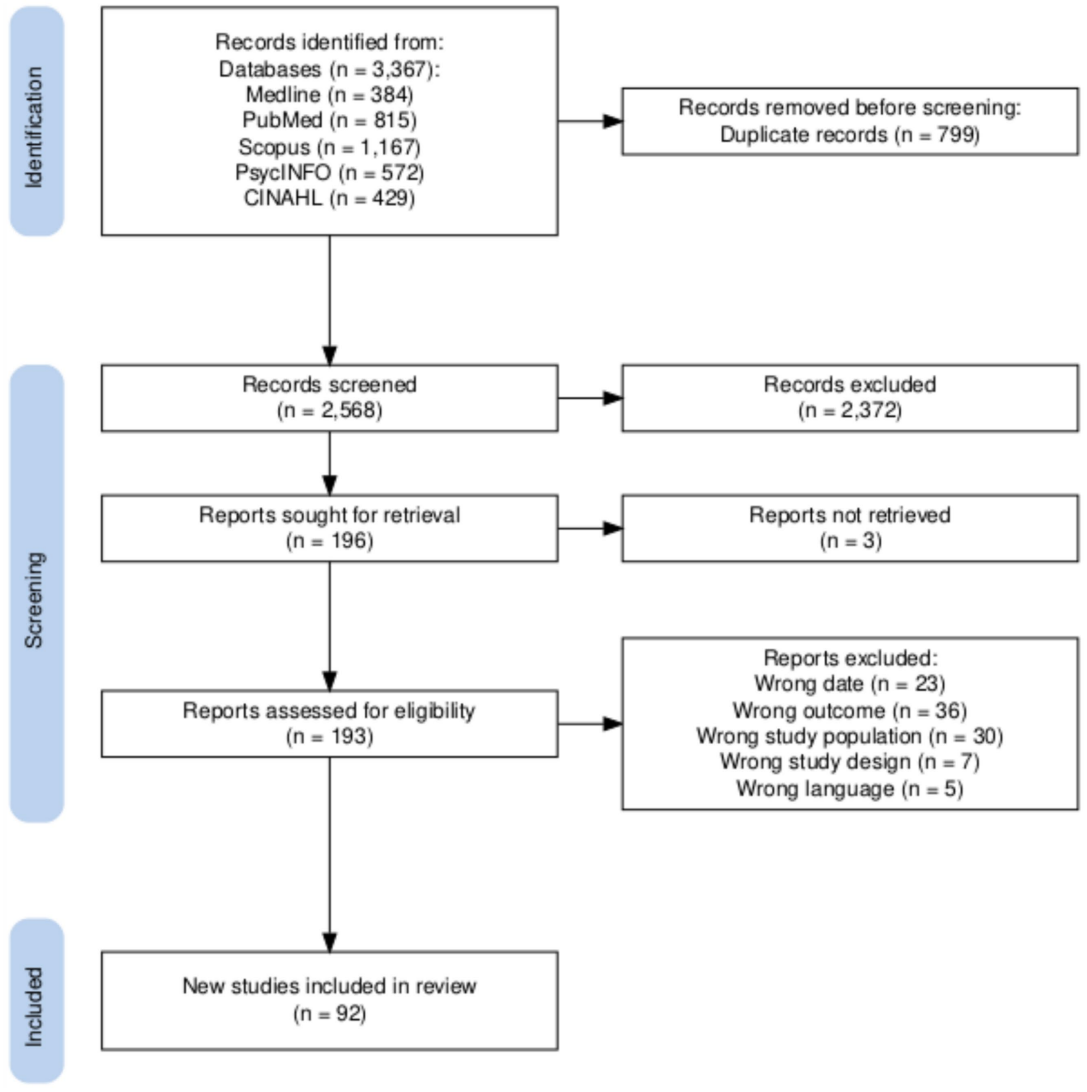
PRISMA flow diagram illustrating the selection process for relevant studies on the prevalence and correlates of burnout, depression, and anxiety among physicians and postgraduate medical trainees.

A total of 3,367 records were retrieved from the search (Figure 1). After removing 799 duplicates, 2,568 publications remained. Title and abstract screening further reduced this number to 196 publications for full-text review. Of these, 3 could not be retrieved, leaving 193 publications for review. Ultimately, 101 articles were excluded, resulting in 92 articles selected for data extraction.

### 3.1 Study characteristics

**Figure 2:**
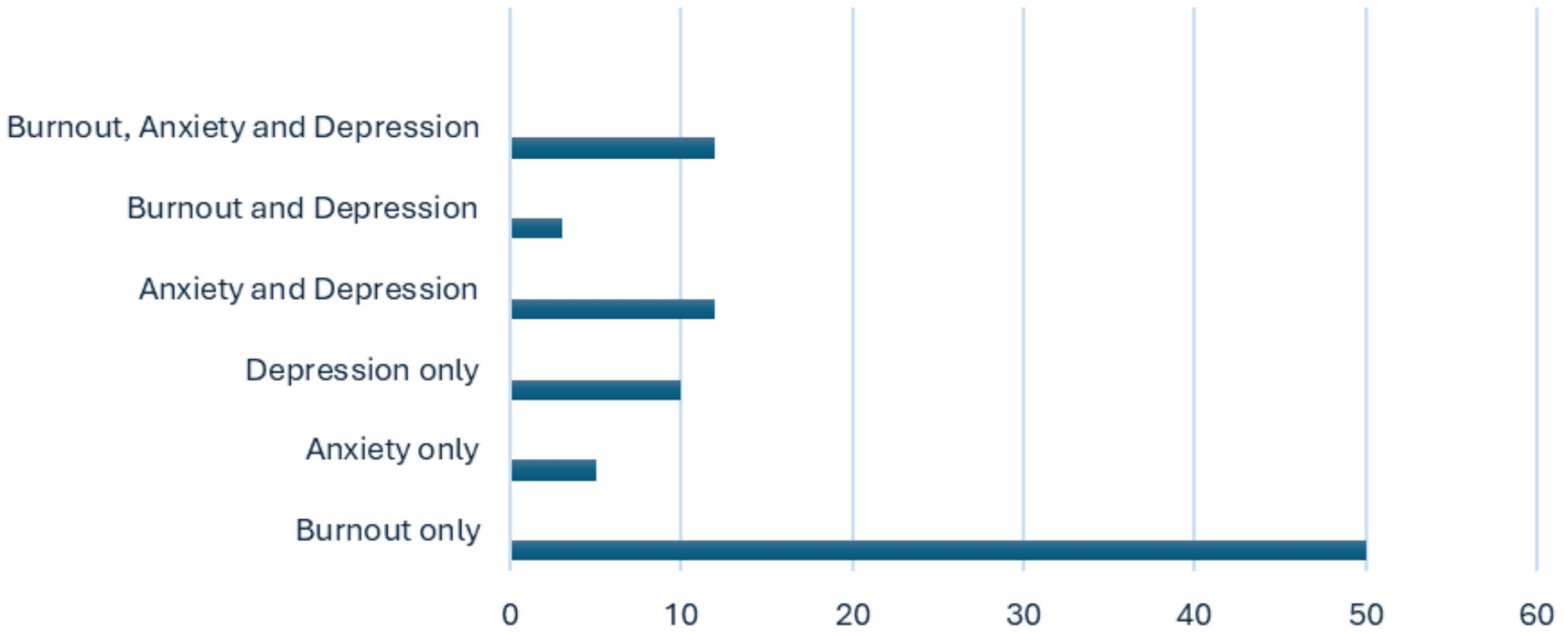
Number of articles reporting burnout, anxiety, depression or combinations of these conditions.

**Figure 3:**
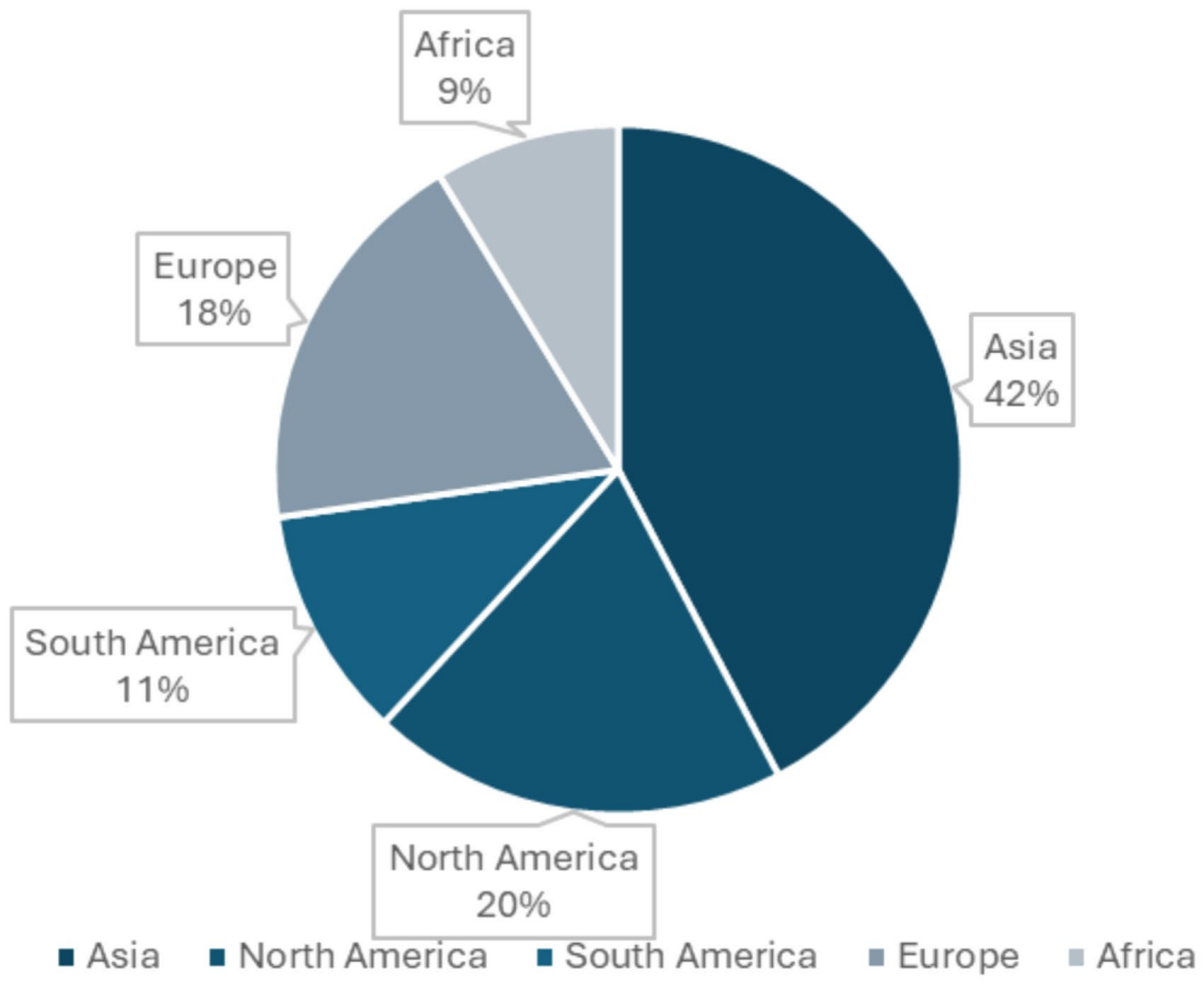
Summary of studies by continents.

The articles reviewed included study designs such as cross-sectional, longitudinal, transverse, cohort and mixed methods. Of the 92 articles included in the review, 44 (47.8%) were published in 2021, 27 (29.3%) were published in 2022, 16 (17.4%) were published in 2023 and 5 (5.4%) were published in 2024. Among the studies, four were cohort studies (43–46), two used mixed methods (47, 48), one was transverse (49), one was longitudinal (50), and the remaining 84 were cross-sectional. The sample sizes ranged from 120 to 11,570 for residents in training and from 51 to 55,000 for physicians/doctors. Out of the 92 studies, 50 focused solely on burnout, 10 addressed only depression, and 5 examined anxiety alone. Additionally, 12 studies investigated both anxiety and depression, 3 focused on burnout and depression, and 12 covered burnout, anxiety, and depression (as shown in Figure 2). Burnout was the most frequently assessed condition 70.65% (*n* = 65), followed by depression 40.2% (*n* = 37) and anxiety 29.3% (*n* = 29). Response rates varied widely from 9.9 to 96.89%, with 22 studies not reporting response rates at all. Most of the studies were conducted in Asia, accounting for 42% (*n* = 39), followed by North America at 20% (*n* = 18), Europe at 18% (*n* = 17), South America at 11% (*n* = 10), and Africa at 9% (*n* = 8) as illustrated in Figure 3. The target population in most studies was physicians 67.4% (*n* = 62), followed by residents 27.2% (*n* = 25), with 5.4% (*n* = 5) targeting both physicians and residents.

### 3.2 Prevalence of burnout

Sixty-five (70.7%) of the included studies addressed burnout (Table 1). Out of these, 76.9% (50 out of 65) focused solely on burnout (45–48, 50–95), 4.6% (3 studies) assessed both burnout and depression (44, 96, 97), and 18.5% (12 studies) examined burnout together with depression, and anxiety (98–109). Of the 65 studies on burnout, 26.2% (*n* = 17) sampled residents, 7.7% (*n* = 5) sampled both residents and physicians, while the remainder 66.2% (*n* = 43) focused on physicians. Most surveys (*n* = 45) used the MBI or a variation of it, and researchers presented the outcomes in different ways (Table 1). Although the majority of studies used the MBI tool, the criteria for classifying ‘overall burnout’ varied. Some studies defined burnout as having at least one of the following: high levels of emotional exhaustion, high depersonalization, or low personal accomplishment (79, 82, 84). Others required high levels in all three constructs simultaneously (71). Other tools included the Copenhagen Burnout Inventory (CBI) (51, 58, 61, 66, 72, 76, 89, 94, 96, 109), Oldenburg Burnout Inventory (OLBI) (87, 106), and the Mini-Z Burnout Assessment (62, 104). Single-study tools included the Burnout Assessment Tool (44), Burnout Measure-Short Version (48), Professional Fulfillment Index (59), Well-Being Index (55), and Professional Quality of Life Scale (46). One study used a single-item, non-proprietary validated burnout measure developed by Schmoldt and colleagues (80). Nearly all studies reported the overall prevalence of burnout, which ranged from 4.7% (44) to 94% (63). For residents, the prevalence ranged from 18.3% (71) to 94% (63), while for physicians, it ranged from 4.7% (44) to 90.1% (61). Burnout prevalence was generally higher among females compared to males, except for two studies which reported the opposite (82, 102). Most of the included studies (*n* = 63/65) identified factors associated with burnout among physicians and residents, while the remaining two papers reported only the prevalence of burnout (84, 107).

### 3.3 Prevalence of depression

A total of 37 studies reported the prevalence of depression among physicians or residents in training (Table 2). Of these, 27% (10 studies) focused solely on depression, 32.4% (12 studies) examined both depression and anxiety, 8.1% (3 studies) looked at depression and burnout, and another 32.4% (12 studies) assessed depression in combination with both burnout and anxiety. Among these studies, 32.4% (12 studies) sampled residents, 5.4% (2 studies) sampled both residents and physicians, and 62.2% (23 studies) focused exclusively on physicians. The study samples varied, as did the tools used to measure depression. The most frequently used tool was the Patient Health Questionnaire, employed in 54.1% (20 studies) of the studies (43, 96, 98, 100, 102, 104–107, 110–120). Other tools included the Depression Anxiety Stress Scale (DASS), used in 16.2% (6 studies) (101, 103, 109, 121–123), the Hospital Anxiety Depression Scale (HADS), used in 13.5% (5 studies) (99, 108, 124–126), and the Center for Epidemiological Studies Depression scale (CESD), used in 5.4% (2 studies) (3, 127). Single-study tools included the Self-Rating Depression Scale (SDS) (128), Symptom Checklist-Core Depression (SCL-CD) (44), General Health Questionnaire (GHQ) (129), and Primary Care Evaluation of Mental Disorders (PRIME-MD) (97). The overall prevalence of depression varied widely, ranging from 4.8% (44) to 66.5% (109) among physicians, and from 7.7% (111) to 93% (119) among residents in training (Table 2). Depression prevalence was generally higher among females compared to males. Most of the studies (*n* = 33) explored factors associated with depression, while the remaining four studies focused solely on prevalence.

**Table 2:** Prevalence and correlates of depression among Physicians and postgraduate medical trainees in studies conducted from 2021 to 2024.

Author’s name	Year of publication	Country study was conducted	Study population	Sample size	Response rate	Tool for measuring burnout	Prevalence of depression level(s)	Associated factors
Appiani et al. (99)	2021	Argentina	Physicians	440	55%	HADS	21.9%	Increasing depression: transient SARS-CoV-2-like symptoms taking anxiolytics working 24-h shifts at the emergency department physicians with less seniority
Jaulin et al. (124)	2021	France	Residents	2,302	22.5%	HADS	7.8%	Increasing depression: female gender working time (volume of hours worked above 60 h per week) on-going training in intensive care
Ouazzani Housni Touhami et al. (110)	2023	Morocco	Doctors	1,267	63.3%	PHQ-9	31.5%	Increasing depression: working in primary and secondary hospitals^*^ moderate and high, stress perceptions^*^ chronic physical illness^*^ family history of psychiatric disorder^*^
Hain et al. (100)	2021	South Africa (SA)	Doctors	213	45%	PHQ-9	35.6%	Associated with depression: doctors planning on leaving the public sector within the next 2 years country of qualification, with SA-qualified doctors reporting higher rates.
Crudden et al. (101)	2023	Ireland	Physicians	2,160	21.9%	DASS	25.8%	Increasing depression: high levels of emotional exhaustion higher clinical workload
de Mélo Silva Júnior et al. (102)	2022	Brazil	Residents	1,989	71.4%	PHQ-4	46.9%	Associated with depression: female sex longer duty hours absence of day off poor learning perception poor feeling about the residency program overall occurrence of psychological abuse anxiety diurnal somnolence and burnout
Alwashmi et al. (103)	2021	Saudi Arabia	Physicians (psychiatrist)	101	Not reported	DASS-21	6.9%	No significant influence on depression
Carlson et al. (98)	2021	USA	Physicians	186	56%	PHQ-2	8%	No information provided
Steil et al. (106)	2022	Brazil	Residents	3,071	10%	PHQ-9	67.7%	Increasing depression: being a woman avoidance of seeing patients with confirmed or suspected cases of COVID-19 working in a wing with a high risk of Contamination the belief that personal protection equipment is not efficacious fear of getting COVID-19 and transmitting it to significant Others having personal relationships impaired since the pandemic
de Mélo Silva Júnior et al. (107)	2023	Brazil	Physician residents	First cohort(pre-COVID): 524 Second cohort (pandemic group): 419	Not reported	PHQ-2	-pre-COVID cohort: 46.0% -pandemic cohort: 58.8%	No information provided
Pogosova et al. (108)	2021	Russia	Physicians	108	Not reported	HADS	22.7%	Increasing depression: being a male physician
Rahimaldeen et al. (109),	2021	Saudi Arabia	Physicians (pediatricians)	386	65%	DASS-21	66.5%	Increasing depression: female gender being junior pediatrician being younger pediatricians
Kuriyama et al. (104)	2022	Japan	Physicians	1,173	18.2%	PHQ-9	15.4%	No information provided
Hasan et al. (125)	2022	Bangladesh	Physicians	442	93.2%	HADS	48.5%	Increasing depression: being female physicians who had experienced COVID-19 like symptoms during the pandemic those who had not received incentives those who used self-funded personal protective equipment (PPE) not received adequate training lacking perceived self-efficacy to manage COVID-19 positive patients greater perceived stress of being infected fear of getting assaulted/humiliated being more connected with social media having lower income levels to support the family feeling more agitated less than 2 h of leisure activity per day short sleep duration.
Rahman et al. (123)	2021	Bangladesh	Physicians	395	Not reported	DASS-21	55.3%	Increasing depression: being a male physician age (physicians less than or equal to 27 years) * physicians with previous history of mental health issues
Carneiro Monteiro et al. (105)	2021	Brazil	Psychiatry residents	185	62%	PHQ-2	16.5%	No information provided
Debnath et al. (122)	2023	Bangladesh	Trainee physicians	130	83%	DASS-21	53.7%	Associated with depression: not receiving mental health counseling during the pandemic anxiety stress loneliness
Pitanupong et al. (111)	2024	Thailand	Psychiatrists/psychiatry trainees	622	36.2%	PHQ-9	Overall prevalence: 12.4% Psychiatrists: 13.9% Psychiatry trainees: 7.7%	Depression in Psychiatrists was associated with: loneliness perceived levels of work satisfaction work stress Depression in Psychiatry trainees was associated with: loneliness perceived level of ability to control work schedule.
Ji et al. (129)	2023	China	Doctors	750	94%	GHQ-12	40.85%	Associated with depression: interaction of long working hours effort-reward imbalance
Chen et al. (43)	2022	China & USA	Resident physicians	China: 3,666 USA: 14,723	China: 45% USA: 56%	PHQ-9	Overall prevalence: China—35.1% USA—34.9%	Associated with depression (USA): neuroticism early family environment female gender not being coupled long duty hours reduced sleep duration Associated with depression (China): young age long duty hours reduced sleep duration
Ng et al. (112)	2021	Hong Kong	Doctors	1,607	393 (24.4%)	PHQ-9	16.0%	Increasing depression: sleeping fewer hours per night
Chen et al. (3)	2022	China	Physicians	15,455	Not reported	CESD-20	35.59%	Increasing depression: female physician^*^ younger age^*^ unmarried^*^ smokers^*^ having a low salary^*^ higher education level^*^ long working tenure^*^ poor health status and sleep quality^*^ history of hypertension and coronary heart disease^*^
Fu et al. (127)	2021	China	Physicians	677	96.89%	CESD-10	42.3%	Increasing depression (both male and female physicians): lower subjective support score lower objective support score Increasing depression (only male physicians): lower support utilization score
Nair et al. (113)	2021	Malaysia	Residents	Estimated to be 448	Estimated to be around 50%	PHQ-9	25.1%	Increasing depression: longer working hours missing meals at work being a resident in the department of surgery and department anaesthesia Decreasing depression: Protected study time having CMEs/lectures leisure or hobby exercise
Khatun et al. (114)	2021	Bangladesh	Physicians	114	Not reported	PHQ-9	34.2%	Increasing depression: being a female* unmarried/divorced/widowed/separated physicians^*^ younger physicians (<35 years) ^*^
Sharp et al. (97)	2021	USA	Fellows	976	51%	PRIME-MD	41%	Increasing depression: financial concern^*^ working more than 70 hours in an average clinical week^*^ the burdens of electronic health record (EHR) documentation^*^
Abu-Elenin (115)	2021	Egypt	Physicians	254	93.36%	PHQ-9	43.8%	Associated with depression: poor sleep quality being a resident physician disrupted social life stigma exposure due to COVID-19
Hagqvist et al. (44)	2022	Sweden	Physicians	6,699	41%	SCL-CD6	4.8%	Increasing depression: being female physicians being junior physicians
He et al. (128)	2021	China	Doctors	1,521	Not reported	SDS	16.9%	Increasing depression: female sex^*^ having a minor child^*^ Decreasing depression: older age^*^
Jarad et al. (116)	2023	Saudi Arabia	Physicians	917	48%	PHQ-9	45.7%	Associated with depression: physicians aged 25–30 years females residents physicians who expressed self-perceived reduction in work quality Independent predictors of depression: female gender^*^ self-perceived reduction in work quality^*^
Bai et al. (117)	2022	China	Residents	1,533	86.48%	PHQ-9	Overall prevalence: 44.9% Moderate/severe symptoms: 12.9%	Increasing depression: poor sleep quality lower optimism of psychological capital higher depersonalization reduced personal accomplishment inappropriate working duration weekly higher emotional exhaustion
Quintana-Domeque et al. (118)	2021	Catalonia (Spain), Italy and UK	Doctors	55,000	First round (June 2020): 3,025 (5.5%) Second round (Nov/Dec 2020): 2,250 (4.1%)	PHQ-9	Overall prevalence: Catalonia: June 2020–17.4%, Nov/Dec 2020–15.9% Italy: June 2020–20.1%, Nov/Dec 2020–21.7% UK-: June 2020–13.7%, Nov/Dec 2020–20.0%	Increasing depression: being a women individuals below 60 years old feeling vulnerable/exposed at work reporting normal/below-normal health.
Kwan et al. (96)	2021	Hong Kong	Doctor/residents	2,879	Doctors-284 (9.9%) Residents-in-training-not reported	PHQ-9	21%	Positively associated with depression: number of working hour(s) per week Negatively associated with depression: Doctors who completed a project-based learning curriculum during undergraduate studies
Hameed et al. (119)	2021	Saudi Arabia	Residents	425	42.6%	PHQ-2	93%	Associated with depression: excessive sleepiness
Elghazally et al. (120)	2021	Egypt	Physicians	2,331	Not reported	PHQ-9	Mild depression: Group 1–31.2% Group 2–32.9% Severe depression: Group 1–5.1% Group 2–14.6%	Increasing depression: females younger age groups divorced or widowed frontline physicians −1–5 years of work experience specialty jobs contact with patients with COVID-19
Sarkar et al. (126)	2021	Bangladesh	Physicians (gastroenterologists)	166	37.9%	HADS	20.7%	Depression was more common in: gastroenterologists of older (41-50-years) age group doing government service service length ≤ 15 years working as specialist less than or equal to 10 years
Varela et al. (121)	2021	Venezuelan	Residents	120	Not reported	DASS-21	11.7%	Associated with depression: marital status (married and divorced residents)

HADS, Hospital Anxiety and Depression Scale; PHQ-9, Patient Health Questionnaire-9; PHQ-4, Patient Health Questionnaire-4; DASS, Depressive Anxiety Stress Scale; GHQ-12, General Health Questionnaire-12; CES-D, Center for Epidemiological Studies Depression scale; PRIME-MD, Primary Care Evaluation of Mental Disorders; SCL-CD6, Symptom Checklist-Core Depression; SDS, Self-Rating Depression Scale.

* Multivariate analysis.

### 3.4 Prevalence of anxiety

A total of 29 studies investigated the prevalence of anxiety among physicians and/or residents in training (Table 3). Among these, 17.2% (5 studies) focused exclusively on anxiety, 41.4% (12 studies) examined both anxiety and depression and another 41.4% (12 studies) assessed anxiety along with burnout and depression. Of these studies, 69% (20 studies) sampled physicians, while 31% (9 studies) focused on residents in training. The most commonly used survey tool is the Generalized Anxiety Disorder scale or its variations, utilized in 48.3% (14 studies) (49, 98, 100, 104, 106, 107, 110, 114–116, 118, 130–132), with outcomes detailed in (Table 3). Other tools included the Depression Anxiety Stress Scale (DASS), used in 20.7% (6 studies) (101, 103, 109, 121–123), and the Hospital Anxiety Depression Scale (HADS), used in 17.4% (5 studies) (99, 108, 124–126). Additionally, single-study tools included the Beck Anxiety Inventory (BAI) (133), the Self-Rating Anxiety Scale (SAS) (128), the Patient Health Questionnaire (PHQ) (102), and the Diagnostic and Statistical Manual of Mental Disorders (DSM-5) (105). The overall prevalence of anxiety ranged from 8% (131) to 78.9% (115) among physicians and from 10% (49) to 63.9% (122) among residents in training. Additionally, the prevalence of anxiety reported in the included studies showed higher levels among females. Most of the studies (*n* = 25) investigated factors associated with anxiety, while the remaining four studies did not provide any information on associated factors with anxiety.

**Table 3:** Prevalence and correlates of anxiety among Physicians and postgraduate medical trainees in studies conducted from 2021 to 2024.

Author’s name	Year of publication	Country study was conducted	Study population	Sample size	Response rate	Tool for measuring anxiety	Prevalence of anxiety level(s)	Associated factors
Appiani et al. (99)	2021	Argentina	Physicians	440	55%	HADS	44%	Increasing anxiety: transient SARS-CoV-2-like symptoms taking anxiolytics working 24-h shifts at the emergency department physicians with less seniority
Jaulin et al. (124)	2021	France	Residents	2,302	22.5%	HADS	19.8%	Increasing anxiety: female gender working time (volume of hours worked above 60 h per week) on-going training in intensive care
Crudden et al. (101)	2023	Ireland	Physicians	2,160	21.9%	DASS	13.8%	Associated with Anxiety: reduced satisfaction with remuneration
Bai et al. (130)	2021	China	Residents	1,533	86.48%	GAD-7 (Chinese version)	Overall prevalence: 32.8% Major anxiety symptoms: 9.9%	Associated with major anxiety: poor sleep Quality^*^ higher emotional Exhaustion^*^ higher depersonalization* reduced personal Accomplishment*
Hain et al. (100)	2021	South Africa	Doctors	213	45%	GAD-7	23.3%	Associated with Anxiety: doctors planning to leave the public sector in the next 2 years. occupational rank
Ouazzani Housni Touhami et al. (110)	2023	Morocco	Doctors	1,267	63.3%	GAD-7	29.2%	Increasing anxiety: being female^*^ working in primary and secondary hospitals^*^ moderate and high-stress perceptions^*^ chronic physical illness^*^ family history of psychiatric disorder*
Alwashmi et al. (103)	2021	Saudi Arabia	Physicians (psychiatrist)	101	Not reported	DASS-21	22.8%	Increasing anxiety: handling COVID-19 patients.
Kuriyama et al. (104)	2022	Japan	Physicians	1,173	18.2%	GAD-7	34.6%	Associated with Anxiety: having no partner^*^ stigma^*^ experience of self-quarantine^*^
Carneiro Monteiro et al. (105)	2021	Brazil	Psychiatry residents	185	62%	DSM-5 Self-Rated Level 1 Cross-Cutting Symptom Measure-Adult	53%	No information provided
Steil et al. (106)	2022	Brazil	Residents	3,071	10%	GAD-7	52.8%	Increasing anxiety: being a woman avoidance of seeing patients with confirmed or suspected cases of COVID-19 failure of supervisor support for the treatment of COVID-19 patients working in a wing with high risk of contamination belief that personal protection equipment is not efficacious fear of getting COVID-19, transmitting it to significant others having personal relationships impaired since the pandemic
de Mélo Silva Júnior et al. (107)	2023	Brazil	Physician residents	First cohort(pre-COVID): 524 Second cohort (pandemic group): 419	Not reported	GAD-2	-pre-COVID cohort: 56.5% -pandemic cohort: 56.5%	No information provided
Pogosova et al. (108)	2021	Russia	Physicians	108	Not reported	HADS	23.8%	Increasing anxiety: being a female physician
Rahimaldeen et al. (109)	2021	Saudi Arabia	Physicians (pediatricians)	386	65%	DASS-21	71.3%	Increasing anxiety: female gender being junior pediatrician being younger pediatricians
Hasan et al. (125)	2022	Bangladesh	Physicians	442	93.2%	HADS	67.72%	Increasing anxiety: being female physicians who had experienced COVID-19 like symptoms during the pandemic those who had not received incentives those who used self-funded personal protective equipment (PPE) not received adequate training lacking perceived self-efficacy to manage COVID-19 positive patients greater perceived stress of being infected fear of getting assaulted/humiliated being more connected with social media having lower income levels to support the family feeling more agitated less than 2 h of leisure activity per day short sleep duration.
Rahman et al. (123)	2021	Bangladesh	Physicians	395	Not reported	DASS-21	35.2%	Increasing anxiety: age (physicians less than or equal to 27 years) ^*^ history of availing or Receiving psychotherapy being a physician of COVID-19 hospitals
Debnath et al. (122)	2023	Bangladesh	Intern Doctors (Trainee physicians)	130	83%	DASS-21	63.9%	Associated with anxiety: depression stress
de Mélo Silva Júnior et al. (102)	2022	Brazil	Residents	1,989	71.4%	PHQ-4	56.6%	Increasing Anxiety: being a woman older age more frequent diurnal somnolence unsatisfactory work-personal life balance depression
Khatun et al. (114)	2021	Bangladesh	Physicians	114	Not reported	GAD-7	32.5%	Increasing anxiety: physicians who worked in Dhaka division physicians who worked more than 8 h per day
Sharma et al. (131)	2021	India	Physicians	100	Not reported	GAD-7	Minimal: 53% Mild: 27% Moderate: 12% Severe: 8%	Associated with anxiety: working in primary or secondary level healthcare facility^*^ sleep disturbance^*^
Abu-Elenin (115)	2021	Egypt	Physicians	254	93.36%	GAD-7	78.9%	Increasing anxiety: poor sleep quality being a resident physician disrupted social life stigma exposure due to COVID-19
He et al. (128)	2021	China	Doctors	1,521	Not reported	SAS	11.11%	Increasing anxiety: female sex^*^ having a minor child*
Saeed et al. (132)	2021	Iraq	Physicians	450	44.7%	GAD-7	Mild: 28.4% Moderate: 39.3% Severe: 22.9%	Associated with anxiety (moderate/severe): working in COVID-19 centers being a general practitioner
Jarad et al. (116)	2023	Saudi Arabia	Physicians	917	48%	GAD-7	43.4%	Associated with anxiety: physicians aged 25–30 years females residents physicians working an average of > 11 h/day physicians reporting self-perceived reduction in work quality Independent predictors of anxiety: female gender^*^ working an average 9–11 h/day^*^ self-perceived reduction in work quality ^*^
Zehra et al. (49)	2022	Pakistan	Residents	260	Not reported	GAD-7	Mild: 35% Moderate: 16.9% Severe: 10.0%	Increasing anxiety: -younger age (mild) -single status (moderate and severe) -low household income (severe) -lack of job satisfaction (severe) Protective towards anxiety: -being male
Quintana-Domeque et al. (118)	2021	Catalonia (Spain), Italy and UK	Doctors	55,000	First round (June 2020): 3,025 (5.5%) Second round (Nov/Dec 2020): 2,250 (4.1%)	GAD-7	Overall prevalence: Catalonia: June 2020–15.9%, Nov/Dec 2020–14.0% Italy: June 2020–24.6%, Nov/Dec 2020–28.2% UK-: June 2020–11.7%, Nov/Dec 2020–17.9%	Increasing anxiety: being a women individuals below 60 years old feeling vulnerable/exposed at work reporting normal/below-normal health.
Chalhub et al. (133)	2021	Brazil	Physicians	450	49.6%	BAI	17%	Associated with anxiety: being female physician burnout (high EE, high DP, and lower PA)
Sarkar et al. (126)	2021	Bangladesh	Physicians (gastroenterologists)	166	37.9%	HADS	25.4%	Associated with anxiety: gastroenterologists of older (41-50-years) age group working as specialists less than or equal to 5 years
Varela et al. (121)	2021	Venezuelan	Residents	120	Not reported	DASS-21	39.2%	No information provided
Carlson et al. (98)	2021	USA	Physicians	186	56%	GAD-2	11%	No information provided

HADS, Hospital Anxiety and Depression Scale; DASS, Depressive Anxiety Stress Scale; GAD-7, Generalized Anxiety Disorder 7-item; GAD-2, Generalized Anxiety Disorder 2-item; PHQ-4, Patient Health Questionnaire-4; SAS, Self-Rating Anxiety Scale; BAI, Beck Anxiety Inventory; DSM-5, Diagnosis and Statistical Manual of Mental Disorders; EE, emotional exhaustion; DP, depersonalization; PA, personal accomplishments.

* Multivariate analysis.

### 3.5 Factors associated with burnout, depression and anxiety

Factors associated with burnout, depression and anxiety were grouped into the following categories: sociodemographic, psychological, social, and organizational. Most of these factors were increasing burnout, depression and anxiety, but protective factors were also identified.

#### 3.5.1 Factors associated with burnout

##### 3.5.1.1 Sociodemographic factors

Age: In eight studies, younger age was associated with higher levels of burnout (51, 54, 57, 61, 78, 79, 102, 109). One study specifically found that younger residents were more likely to experience reduced personal accomplishment (PA) (77). The impact of older age on burnout was less consistent: three studies reported that older individuals experienced lower levels of burnout (46, 64, 96), while another study found higher burnout rates among older age groups (50).

Gender: Sixteen studies found that females experienced higher levels of burnout (45, 46, 48, 51, 54, 58, 61, 69, 70, 76, 79, 89, 100, 103, 108, 109). Conversely, two studies reported that males had higher burnout levels (82, 102).

Marital Status/Having Children: The findings on marital status and burnout were inconsistent. In some studies, being married was associated with increased burnout (82, 104), while in others, it was linked to decreased burnout (61). Being single or not married was associated with higher burnout levels (46). Additionally, four studies found that having children increased burnout (50, 60, 61, 82), whereas one study reported that having more children served as a protective factor against burnout (65).

Financial Situation: Factors such as inadequate compensation (94), financial problems (72), financial pressure (45), lower income (87), and educational debt exceeding $250,000 (69) were all associated with increased burnout. Conversely, financial well-being was linked to decreased burnout (61), and higher income satisfaction was identified as a protective factor against burnout (65).

Professional Experience: Three studies found increased burnout among junior physicians (44, 99, 109). Being a resident was often associated with increased burnout (51, 85, 87, 103), though one study reported decreased burnout (68). Less professional experience generally correlated with higher burnout (60, 61, 75, 81, 89).

##### 3.5.1.2 Psychological factors

Higher burnout was associated with pre-existing psychological factors including depression (54, 56, 58, 68, 101, 102), anxiety (54, 56, 58), and stress (66, 78).

##### 3.5.1.3 Social factors associated with burnout

Burnout was associated with several social factors, including psychological abuse (102), unfair treatment at work (55), poor work-life balance and lack of vacation or leisure (57), limited social activities (67), stigmatization for treating COVID-19 patients, and workplace violence (72). In four studies, physicians and residents reported that family life was associated with increased burnout. High burnout was linked to factors such as the quality of family relationships (105), living with a family member with comorbidities (61), limited family time for residents (93), and strained personal relationships since the COVID-19 pandemic (106).

##### 3.5.1.4 Organizational factors

Eight studies found that working long hours (over 40 h per week) were associated with higher burnout (67, 68, 77, 78, 82, 89, 93, 97). Additionally, more frequent night shifts (48, 88), extended on-call hours (54, 88), and 24-h shifts (99) were all linked to increased burnout.

##### 3.5.1.5 Burnout related to COVID-19 pandemic

The COVID-19 pandemic led to higher burnout due to factors including transient symptoms (99), caring for COVID-19 patients (53), fear of infection, working in high-risk contamination areas, concerns about PPE effectiveness (106), and testing positive for COVID-19 (48).

##### 3.5.1.6 Protective factors against burnout

The authors also highlighted protective factors against burnout, including resilience and strong institutional support (56), having more children and greater income satisfaction (65), and faculty support (93). Additionally, researchers identified several other factors that help reduce burnout: professional efficacy (101), access to mental health services and insurance for personal illness or emergencies (97), staff grade or trainee status combined with higher perceived support (68), being of Black or Asian descent and being in smaller programs (69), being an International Medical Graduate (IMG) and part of a racial minority (74), and regular exercise (three times a week for 20 min) (46).

#### 3.5.2 Factors associated with depression

##### 3.5.2.1 Sociodemographic factors

Age: Younger age was linked to higher levels of depression in 7 studies (3, 43, 109, 114, 116, 120, 123). The relationship between older age and depression was inconsistent. One study found that older age was associated with lower depression rates in a multivariable analysis (128), while another study found the opposite, with older age linked to higher depression (126).

Gender: Thirteen studies identified being female as a factor associated with increased depression (3, 43, 44, 102, 106, 109, 114, 116, 118, 120, 124, 125, 128), while two studies reported higher depression rates in males (108, 123).

Marital Status/Having Children: In four studies, being single or unmarried was associated with higher depression (3, 43, 114, 120). Only one study found that being married was linked to increased depression (121). Additionally, a multivariate analysis indicated that having children was associated with higher depression levels (128).

Educational Level and Financial Situation: A multivariate analysis found that a higher educational level was linked to increased depression (3). Low income (125), low salary (3), and financial concerns (97) were associated with higher depression.

##### 3.5.2.2 Professional experience

Two studies reported increased depression among junior physicians (44, 99). Three studies found that being a resident in training was linked to increased depression (113, 115, 116), and less professional experience was associated with higher depression (120).

##### 3.5.2.3 Psychological factors

Higher depression levels were associated with pre-existing psychological factors, including anxiety (102, 122), burnout (102), stress (110, 111, 122), and poor sleep (3, 43, 115, 117, 125).

##### 3.5.2.4 Social factors

Several social factors were linked to increased depression, including psychological abuse (102), stigmatization from exposure to COVID-19 and disrupted social life (115), and fewer than 2 h of daily leisure activities (125). In one study, having a hobby or leisure time was associated with lower depression (113).

##### 3.5.2.5 Organizational factors

Four studies found that long working hours were associated with higher depression (43, 97, 113, 124). Additionally, working 24-h shifts in the emergency department (99) and a higher clinical workload (101) were all linked to increased depression.

##### 3.5.2.6 Depression related to the COVID-19 pandemic

The COVID-19 pandemic led to increased depression levels due to several factors, including transient symptoms (99, 125), direct contact with COVID-19 patients (120), avoiding patients with confirmed or suspected COVID-19 cases, working in high-risk contamination areas, fear of contracting the virus and transmitting it to loved ones (106); and a lack of confidence in effectively managing COVID-19 patients (125).

#### 3.5.3 Factors associated with anxiety

##### 3.5.3.1 Sociodemographic factors

Age: Four studies (49, 109, 116, 123) found that younger age was linked to higher levels of anxiety. Conversely, two studies (102, 126) found that older age was associated with increased anxiety.

Gender: Eleven studies (102, 106, 108–110, 116, 118, 124, 125, 128, 133) identified being female as a factor associated with increased anxiety, while one study (49) reported that being male was a protective factor against anxiety.

Marital Status/Having Children: Two studies (49, 104) linked being single or unmarried with higher anxiety. Additionally, a multivariate analysis suggested that having children was associated with higher anxiety levels (128).

Financial Situation: Factors such as lower income levels (125), dissatisfaction with remuneration (101), and low household income (49) were all linked to higher anxiety.

##### 3.5.3.2 Occupational and professional experience

Two studies (99, 109) reported increased anxiety among junior physicians while being a resident was associated with higher anxiety in two studies (115, 116). Increased anxiety was also linked to a lack of job satisfaction (49) and working as a specialist for 5 years or less (126).

##### 3.5.3.3 Psychological factors

Anxiety was associated with stress (122, 125), depression (102, 122), burnout (133), and poor sleep (115, 125, 130, 131). A multivariate analysis linked moderate to high stress perceptions and a family history of psychological disorders to increased anxiety (110).

##### 3.5.3.4 Social factors

Several social factors, such as stigmatization from COVID-19 exposure (104, 115), disrupted social life (115), less than 2 h of daily leisure activities (125), and unsatisfactory work-life balance (102), were associated with increased anxiety.

##### 3.5.3.5 Organizational factors

Increased anxiety was found in physicians working more than 8 h per day (114) or an average of 11 h per day (116), and working 24-h shifts in the emergency department (99). Increased anxiety was reported in residents working over 60 h per week (124).

##### 3.5.3.6 Anxiety related to the COVID-19 pandemic

The COVID-19 pandemic increased anxiety levels due to various factors, including transient symptoms (99, 125); handling of COVID-19 patients (103); avoiding contact with confirmed or suspected COVID-19 cases, working in high-risk areas, fear of contracting and transmitting the virus (106); lack of confidence in managing COVID-19 patients (125); and working in COVID-19 hospitals or centers (123, 132).

## 4 Discussion

The prevalence and correlates of burnout, depression, and anxiety among physicians and postgraduate medical trainees are critical areas of research that have gained significant attention in recent years. This scoping review highlights the alarming rates of mental health issues in this demographic, with burnout, depression, and anxiety being prevalent and deeply intertwined. In this review, the studies varied considerably in their methodology and findings. The most used tools by researchers were the MBI for burnout, the Patient Health Questionnaire-9 (PHQ-9) for depression, and the Generalized Anxiety Disorder-7 (GAD-7) for anxiety. These tools are recognized as the standard instruments to measure these mental health conditions. For burnout, different versions of the MBI were applied. Additionally, even in studies that utilized the same MBI version, results were reported inconsistently. For example, some studies presented burnout rates as an overall figure (55, 100, 103, 106), while others broke down the results into burnout subdimensions (71, 95, 108). Similarly, in studies measuring depression and anxiety, alongside the commonly used PHQ-9 and GAD-7, other instruments such as the Depression, Anxiety, and Stress Scales (DASS) and the Hospital Anxiety and Depression Scale (HADS) were also employed. Several sociodemographic, psychological, social, and organizational factors contribute to these mental health challenges, particularly during the ongoing COVID-19 pandemic. The review indicated a stronger focus on assessing burnout, depression, and anxiety among physicians compared to residents in training. This discrepancy was further evident in studies that included both groups, with physicians being more frequently sampled (60, 85). One potential reason for this could be the differing accessibility between physicians and residents. Physicians often remain in one facility, while residents frequently rotate through different healthcare centers, making it more challenging to reach them for surveys and assessments. The findings of this review underline the urgency of addressing these issues and providing effective interventions and support for healthcare professionals.

### 4.1 Prevalence of burnout, depression, and anxiety

The prevalence of burnout, depression, and anxiety among physicians and postgraduate medical trainees in the included studies ranged widely. For burnout, the review uncovered considerable variability in prevalence estimates among physicians and residents, with significant differences in how burnout was defined and measured across studies. Burnout prevalence ranged widely from 4.7 to 90.1% among physicians and from 18.3 to 94% among residents in training, with higher rates generally found in residents compared to practicing physicians. These findings seem to agree with what has been reported in a previous systematic review (0 to 80.5%) (28). Although global estimates suggest that burnout affects around 50% of both physicians (134) and residents (135), the review found that over 20 studies on physicians and 7 studies on residents reported burnout prevalence levels exceeding 50%. Burnout was most commonly measured using the MBI, although different tools and criteria for burnout classification led to variability in findings. The high prevalence of burnout among residents, in particular, aligns with previous studies that have highlighted the intensity of training, long working hours, and high emotional demands as key contributors (34). The prevalence of depression among physicians and residents also varied significantly, ranging from 4.8 to 66.5% among physicians and 7.7 to 93% among residents. The findings are consistent with prior research indicating that medical trainees and physicians are at heightened risk of depression compared to the general population (15, 17). Anxiety, similarly, had a wide prevalence range, from 8 to 78.9% among physicians, and from 10 to 63.9% among residents. This reflects the intense work pressure, substantial workloads, extended shifts, resource constraints, and organizational changes, all contributing (10–12), to increase mental health issues in physicians and medical trainees. Most of the studies reviewed reported prevalence exceeding 35% in both conditions, which is higher than the 20.5% for depression and 25.8% for anxiety found in a global systematic review and meta-analysis (136). Similarly, Mata et al. reported a 28.8% prevalence of depression among resident physicians (18), a figure lower than what was found in the majority of studies assessing depression in residents included in this review. This lower prevalence of depression reported in Mata et al.’s review compared to most studies included in our analysis may be attributed to several factors: the inclusion of more recent studies that reflect heightened mental health challenges during the COVID-19 pandemic; broader geographic coverage, especially from low- and middle-income countries with diverse healthcare contexts; and methodological variations such as differences in assessment tools, diagnostic thresholds, and sampling strategies.

### 4.2 Sociodemographic factors

Sociodemographic factors, such as age, gender, marital status, and financial situation, were consistently associated with higher levels of burnout, depression, and anxiety. Younger age, particularly among residents and junior physicians, was frequently linked to higher levels of these mental health issues. This is in line with research indicating that early-career professionals are more vulnerable to the psychological stressors of medical training (137). Conversely, the impact of age on burnout and depression in older physicians was inconsistent, suggesting that other factors might influence the relationship between age and mental health outcomes in healthcare professionals. Junior physicians and residents experience higher burnout, anxiety and depression rates (44, 51, 85), likely due to their lack of experience and the overwhelming demands of their roles compared to senior physicians who have acquired experience in the job. Frequent night shifts (48) and extended on-call hours (54, 88), which are more common among junior physicians and residents, further contributing to higher burnout in these groups.

Gender differences were another notable finding. Female physicians and residents generally reported higher levels of burnout, depression, and anxiety compared to their male counterparts (76, 79, 108, 109, 116, 118, 125). This disparity may be explained by gender role theory which suggests that women are more likely to express emotional and physical exhaustion, leading to higher scores on emotional exhaustion scales (138). Also, the added pressure of balancing professional responsibilities and family duties can lead to emotional exhaustion and increased burnout and psychological issues. Additionally, some researchers propose that men may generally exhibit higher resilience (139), which refers to the ability to adapt effectively in the face of stress and adversity (140). Research also suggests that resilience has an inverse relationship with burnout (141), meaning individuals with higher resilience may be better protected against burnout. However, a small number of studies reported higher burnout or depression rates in males, possibly due to cultural or institutional factors that present unique challenges for men.

The relationship between marital status, having children, and mental health outcomes was less consistent. While some studies found that being married or having children increased burnout, depression, and anxiety (60, 82, 104, 121, 128), others identified these factors as protective. The diversity of findings may be due to the complex interaction between personal, professional, and societal expectations, which may differ across cultural and institutional settings.

Financial stress was a significant factor in the mental health challenges faced by physicians and residents. Studies have shown that inadequate compensation, educational debt, and financial insecurity and pressures contribute to burnout, depression, and anxiety (45, 69, 94, 125). A study in South Africa found that lower income was associated with higher burnout levels among physicians during the COVID-19 pandemic (87). These findings highlight the importance of addressing financial well-being as part of broader efforts to improve mental health outcomes in the medical profession.

### 4.3 Psychological and social factors

Psychological factors such as depression, anxiety, and stress were strongly associated with burnout, depression, and anxiety. Physicians and residents with pre-existing psychological conditions are at greater risk of experiencing burnout, anxiety and depression (56, 66, 102). This is not surprising given the interrelated nature of these mental health conditions, which often co-occur among healthcare professionals. A study conducted in Morocco linked high anxiety levels to increased stress perception and a family history of psychological disorders (110). The impact of psychological distress is compounded by the high demands of medical practice, where the emotional toll of patient care and the expectation of constant performance can exacerbate existing mental health struggles.

Social factors, including poor work-life balance, limited social activities, and family life stress, were also significant contributors to burnout, depression, and anxiety. The disruption of social life and family dynamics due to long working hours, night shifts, and emotional exhaustion may lead to healthcare professionals’ mental health issues. Shift work often disrupts work-life balance and contributes to sleep deprivation, further increasing burnout risk (142). In Brazil, residents with an unsatisfactory work-life balance experience higher anxiety levels (102). Conversely, a study in Malaysia found that having hobbies or leisure activities was linked to lower depression levels among residents (113). The COVID-19 pandemic further intensified these social stressors, with many physicians and residents reporting additional challenges such as stigmatization for treating COVID-19 patients (73), family concerns, and fear of infecting loved ones (143).

### 4.4 Organizational factors

Work-related factors, including long working hours, high workload, night shifts, and extended on-call hours, were identified as significant predictors of burnout, depression, and anxiety (67, 68, 77, 99). These findings are consistent with a large body of literature that highlights the detrimental effects of work-related stressors on healthcare workers’ mental health (141). The strain of working over 40 h a week, frequent night shifts, and 24-h shifts exacerbates feelings of exhaustion, stress, and emotional depletion, leading to higher levels of burnout anxiety and depression.

The COVID-19 pandemic has been a key organizational factor in exacerbating these mental health issues. Healthcare workers, particularly those in high-risk areas such as emergency departments and intensive care units (99), reported increased levels of burnout, depression, and anxiety due to the overwhelming demands of treating COVID-19 patients, fear of infection, and inadequate protective measures. The pandemic’s impact on mental health highlights the urgent need for better institutional support, improved personal protective equipment (PPE), and mental health resources for frontline healthcare workers.

### 4.5 Protective factors

Several protective factors were identified in the studies reviewed, including resilience, and strong institutional support (56), access to mental health services (97), and faculty support (93). These findings suggest that fostering a supportive work environment, promoting mental health resources, and encouraging work-life balance can help mitigate the negative impact of stressors on physicians and residents. Additionally, personal factors such as exercising three times a week for 20 min (46), professional efficacy (101), and having a supportive family life were all identified as protective factors against burnout. These findings emphasize the importance of a multifaceted approach to addressing mental health in the medical profession.

### 4.6 Implications for policy and practice

The findings of this scoping review underscore the widespread and significant mental health challenges faced by physicians and postgraduate medical trainees, including burnout, depression, and anxiety. These issues are not only detrimental to the well-being of healthcare providers but also have serious implications for patient care, workforce sustainability, and healthcare system efficiency. Therefore, urgent attention and targeted interventions are required at multiple levels to mitigate the impact of these mental health conditions. Firstly, healthcare organizations must prioritize mental health and well-being in their workplace policies. This includes promoting a culture of psychological safety, providing access to mental health services, and ensuring that physicians and trainees have opportunities to engage in stress-reducing activities. Implementing institutional support systems, such as counseling services, peer support programs, and resilience training, could significantly help reduce burnout and foster a healthier work environment. Reducing work-related demands and enhancing access to resources may help residents lower their stress levels and improve their overall well-being (144).

Furthermore, providing financial support and improving compensation for healthcare professionals, especially in regions with significant income disparities, may help alleviate stressors contributing to these mental health issues. Secondly, addressing work-life balance is critical in both policy and practice. Policies that regulate working hours, reduce excessive shifts, and prevent burnout-inducing workloads should be implemented. For instance, limiting mandatory on-call hours and advocating for reasonable shift schedules, stress management, training in mindfulness could mitigate the stress and burnout identified in this review (145, 146). Additionally, providing sufficient time off and family leave would help professionals manage personal responsibilities alongside demanding work schedules. Finally, the incorporation of mental health education into medical training is essential. Training future healthcare providers to recognize the early signs of burnout, depression, and anxiety, and equipping them with coping strategies, could significantly reduce the prevalence of these conditions in the long term. Integrating mental health discussions into residency and ongoing professional development programs may help destigmatize these issues and empower healthcare providers to seek help when needed.

## 5 Strength and limitations

One of the key strengths of this study is its comprehensive and up-to-date examination of the prevalence of major mental health conditions, such as burnout, depression, and anxiety, which are often studied individually but not collectively. The findings offer valuable data that can assist in monitoring changes in these conditions over time. However, the study has some limitations. Firstly, while a significant number of papers were included, the search did not cover all available databases, leaving a possibility that some relevant studies were missed or excluded due to publication bias. Secondly, the research was limited to studies published between 2021 and April 2024 and written only in English, which further restricts its scope. Thirdly, different methods were used to measure the prevalence of burnout, depression, and anxiety, making it difficult to produce a unified estimate for each condition. Future studies should focus on reporting rates specific to each assessment tool rather than merging results from different scales. Fourthly, many of the studies did not mention the validity and reliability of the tools they used. Among the most used tools were the MBI for burnout, the PHQ-9 for depression, and the GAD-7 for anxiety. These tools are widely used globally, with strong evidence supporting their reliability and consistency. For instance, the GAD-7 demonstrates good test–retest reliability and strong internal consistency (147, 148). The MBI, a concise questionnaire used to evaluate burnout symptoms and their intensity, has shown strong reliability. Specifically, it has Cronbach’s alpha values of 0.90 for emotional exhaustion, 0.76 for depersonalization, and 0.76 for personal accomplishment (149). Similarly, the PHQ-9, widely used for depression screening, exhibits solid psychometric properties with good sensitivity and high internal consistency (150, 151), making it a reliable tool for assessing depression symptoms. Thus, the choice and selection of tools in the retrieved studies seem to be appropriate. Lastly, another limitation of our study is the lack of a formal assessment of bias and methodological quality among the included studies. Future updates to this review will address this gap by incorporating a meta-analysis and employing standardized tools to systematically evaluate the risk of bias and study quality. Despite these limitations, this study provides a crucial resource for future research on the prevalence of burnout, depression, and anxiety, emphasizing the need for consistent methodologies and longitudinal studies.

## 6 Conclusion

The high prevalence of burnout, depression, and anxiety among physicians and postgraduate medical trainees is a concerning issue that requires immediate attention. This review highlights the complex interplay of sociodemographic, psychological, social, and organizational factors contributing to mental health challenges in this group. The psychological well-being of these professionals is critical, as it directly impacts patient care and overall healthcare outcomes. Additionally, our review highlights a consistently high prevalence of burnout, anxiety, and depression across multiple high-quality studies, underscoring the need for urgent action at both policy and institutional levels. To mitigate these issues, healthcare organizations must prioritize the mental health and well-being of their staff by implementing policies that promote work-life balance, financial security, mental health resources, and institutional support. Additionally, addressing the unique challenges faced by female physicians, junior physicians, and residents is essential to creating a healthier and more sustainable medical workforce. Ultimately, improving mental health outcomes in healthcare professionals will lead to better care for patients and a more resilient healthcare system. It is essential to prioritize and implement interventions that support the psychological well-being of physicians and residents, with the goal of preventing or reducing burnout, depression, and anxiety. One promising approach is the use of evidence-based mobile text messaging technology, which offers a convenient, cost-effective, and accessible way to provide psychological support to those in need (152, 153). This review offers valuable insights to inform policymakers and healthcare administrators in designing effective strategies to mitigate burnout, depression, and anxiety among medical professionals.

## Data Availability

The original contributions presented in the study are included in the article/Supplementary material, further inquiries can be directed to the corresponding author.
