# Supplementary material for: Prevalence and correlates of depression, anxiety, and burnout among physicians and postgraduate medical trainees: a scoping review of recent literature
Source: Front Public Health. 2025 Jul 8;13:1537108. doi: 10.3389/fpubh.2025.1537108 (PMC12279716; doi:10.3389/fpubh.2025.1537108)
Supplement: Supplementary file 1 [file Table_1.docx]

**Appendix 1**

**Search Strategy**

We searched Medline, PubMed, Scopus, PsycINFO, and CINAHL. Search terms include: “prevalence of depression,” “prevalence of anxiety,” “prevalence of burnout,” “depression”, “burnout”, “anxiety”, “prevalence”, “physicians,” “doctors,” “medical practitioners,” and “resident physicians.” Search was conducted in April, 2024.

**Example of search in Medline**

1. *Depression/ or depression.mp.

2. Burnout, Psychological/ or Burnout, Professional/ or burnout.mp.

3. Anxiety.mp. or Anxiety/

4. Prevalence/ or prevalence.mp.

5. Physicians, Family/ or Physicians/ or physicians.mp. or Physicians, Primary Care/

6. medical practitioners.mp.

7. Physicians/ or Doctors.mp.

8. "Internship and Residency"/ or resident physicians.mp.

9. 1 and 2 and 3

10. 4 and 9

11. 5 or 6 or 7 or 8

12. 10 and 11

**Example of search in PubMed**

Search: **((((depression) AND (burnout)) AND (anxiety)) AND (prevalence)) AND ((((physicians) OR (doctors)) OR (medical practitioners)) OR (resident physicians))**

("depressed"[All Fields] OR "depression"[MeSH Terms] OR "depression"[All Fields] OR "depressions"[All Fields] OR "depression s"[All Fields] OR "depressive disorder"[MeSH Terms] OR ("depressive"[All Fields] AND "disorder"[All Fields]) OR "depressive disorder"[All Fields] OR "depressivity"[All Fields] OR "depressive"[All Fields] OR "depressively"[All Fields] OR "depressiveness"[All Fields] OR "depressives"[All Fields]) AND ("burnout s"[All Fields] OR "burnout, psychological"[MeSH Terms] OR ("burnout"[All Fields] AND "psychological"[All Fields]) OR "psychological burnout"[All Fields] OR "burnout"[All Fields] OR "burnouts"[All Fields]) AND ("anxiety"[MeSH Terms] OR "anxiety"[All Fields] OR "anxieties"[All Fields] OR "anxiety s"[All Fields]) AND ("epidemiology"[MeSH Subheading] OR "epidemiology"[All Fields] OR "prevalence"[All Fields] OR "prevalence"[MeSH Terms] OR "prevalance"[All Fields] OR "prevalences"[All Fields] OR "prevalence s"[All Fields] OR "prevalent"[All Fields] OR "prevalently"[All Fields] OR "prevalents"[All Fields]) AND ("physician s"[All Fields] OR "physicians"[MeSH Terms] OR "physicians"[All Fields] OR "physician"[All Fields] OR "physicians s"[All Fields] OR ("doctor s"[All Fields] OR "doctoral"[All Fields] OR "doctorally"[All Fields] OR "doctorate"[All Fields] OR "doctorates"[All Fields] OR "doctoring"[All Fields] OR "physicians"[MeSH Terms] OR "physicians"[All Fields] OR "doctor"[All Fields] OR "doctors"[All Fields]) OR (("medic"[All Fields] OR "medical"[All Fields] OR "medicalization"[MeSH Terms] OR "medicalization"[All Fields] OR "medicalizations"[All Fields] OR "medicalize"[All Fields] OR "medicalized"[All Fields] OR "medicalizes"[All Fields] OR "medicalizing"[All Fields] OR "medically"[All Fields] OR "medicals"[All Fields] OR "medicated"[All Fields] OR "medication s"[All Fields] OR "medics"[All Fields] OR "pharmaceutical preparations"[MeSH Terms] OR ("pharmaceutical"[All Fields] AND "preparations"[All Fields]) OR "pharmaceutical preparations"[All Fields] OR "medication"[All Fields] OR "medications"[All Fields]) AND ("practitioner"[All Fields] OR "practitioner s"[All Fields] OR "practitioners"[All Fields])) OR (("internship and residency"[MeSH Terms] OR ("internship"[All Fields] AND "residency"[All Fields]) OR "internship and residency"[All Fields] OR "residencies"[All Fields] OR "residency"[All Fields] OR "reside"[All Fields] OR "resided"[All Fields] OR "residence"[All Fields] OR "residence s"[All Fields] OR "residences"[All Fields] OR "residency s"[All Fields] OR "resident"[All Fields] OR "resident s"[All Fields] OR "residents"[All Fields] OR "resides"[All Fields] OR "residing"[All Fields]) AND ("physician s"[All Fields] OR "physicians"[MeSH Terms] OR "physicians"[All Fields] OR "physician"[All Fields] OR "physicians s"[All Fields])))

**Translations**

**depression:** "depressed"[All Fields] OR "depression"[MeSH Terms] OR "depression"[All Fields] OR "depressions"[All Fields] OR "depression's"[All Fields] OR "depressive disorder"[MeSH Terms] OR ("depressive"[All Fields] AND "disorder"[All Fields]) OR "depressive disorder"[All Fields] OR "depressivity"[All Fields] OR "depressive"[All Fields] OR "depressively"[All Fields] OR "depressiveness"[All Fields] OR "depressives"[All Fields]

**burnout:** "burnout's"[All Fields] OR "burnout, psychological"[MeSH Terms] OR ("burnout"[All Fields] AND "psychological"[All Fields]) OR "psychological burnout"[All Fields] OR "burnout"[All Fields] OR "burnouts"[All Fields]

**anxiety:** "anxiety"[MeSH Terms] OR "anxiety"[All Fields] OR "anxieties"[All Fields] OR "anxiety's"[All Fields]

**prevalence:** "epidemiology"[Subheading] OR "epidemiology"[All Fields] OR "prevalence"[All Fields] OR "prevalence"[MeSH Terms] OR "prevalance"[All Fields] OR "prevalences"[All Fields] OR "prevalence's"[All Fields] OR "prevalent"[All Fields] OR "prevalently"[All Fields] OR "prevalents"[All Fields]

**physicians:** "physician's"[All Fields] OR "physicians"[MeSH Terms] OR "physicians"[All Fields] OR "physician"[All Fields] OR "physicians's"[All Fields]

**doctors:** "doctor's"[All Fields] OR "doctoral"[All Fields] OR "doctorally"[All Fields] OR "doctorate"[All Fields] OR "doctorates"[All Fields] OR "doctoring"[All Fields] OR "physicians"[MeSH Terms] OR "physicians"[All Fields] OR "doctor"[All Fields] OR "doctors"[All Fields]

**medical:** "medic"[All Fields] OR "medical"[All Fields] OR "medicalization"[MeSH Terms] OR "medicalization"[All Fields] OR "medicalizations"[All Fields] OR "medicalize"[All Fields] OR "medicalized"[All Fields] OR "medicalizes"[All Fields] OR "medicalizing"[All Fields] OR "medically"[All Fields] OR "medicals"[All Fields] OR "medicated"[All Fields] OR "medication's"[All Fields] OR "medics"[All Fields] OR "pharmaceutical preparations"[MeSH Terms] OR ("pharmaceutical"[All Fields] AND "preparations"[All Fields]) OR "pharmaceutical preparations"[All Fields] OR "medication"[All Fields] OR "medications"[All Fields]

**practitioners:** "practitioner"[All Fields] OR "practitioner's"[All Fields] OR "practitioners"[All Fields]

**resident:** "internship and residency"[MeSH Terms] OR ("internship"[All Fields] AND "residency"[All Fields]) OR "internship and residency"[All Fields] OR "residencies"[All Fields] OR "residency"[All Fields] OR "reside"[All Fields] OR "resided"[All Fields] OR "residence"[All Fields] OR "residence's"[All Fields] OR "residences"[All Fields] OR "residency's"[All Fields] OR "resident"[All Fields] OR "resident's"[All Fields] OR "residents"[All Fields] OR "resides"[All Fields] OR "residing"[All Fields]

**physicians:** "physician's"[All Fields] OR "physicians"[MeSH Terms] OR "physicians"[All Fields] OR "physician"[All Fields] OR "physicians's"[All Fields]

**Example of search in Scopus**

(TITLE-ABS-KEY (prevalence AND of AND depression) OR TITLE-ABS-KEY (prevalence AND of AND anxiety) OR TITLE-ABS-KEY (prevalence AND of AND burnout) AND TITLE-ABS-KEY (physicians) OR TITLE-ABS-KEY (medical AND doctors) OR TITLE-ABS-KEY (medical AND practitioners) OR TITLE-ABS-KEY (resident AND physicians))

**Example of search in CINAHL**

| **Search ID#** | **Search Terms** | **Search Options** |
| --- | --- | --- |
| S10 | S7 AND S8 | **Limiters** - Publication Date: 20210101-20241231  **Expanders** - Apply related words; Apply equivalent subjects  **Search modes** – Proximity |
| S9 | S7 AND S8 | **Expanders** - Apply related words; Apply equivalent subjects  **Search modes** - Proximity |
| S8 | S4 OR S5 OR S6 | **Expanders** - Apply related words; Apply equivalent subjects  **Search modes** - Proximity |
| S7 | S1 OR S2 OR S3 | **Expanders** - Apply related words; Apply equivalent subjects  **Search modes** - Proximity |
| S6 | resident physicians | **Expanders** - Apply related words; Apply equivalent subjects  **Search modes** - Proximity |
| S5 | medical practitioners | **Expanders** - Apply related words; Apply equivalent subjects  **Search modes** - Proximity |
| S4 | physicians or doctors or clinicians | **Expanders** - Apply related words; Apply equivalent subjects  **Search modes** - Proximity |
| S3 | prevalence of anxiety | **Expanders** - Apply related words; Apply equivalent subjects  **Search modes** - Proximity |
| S2 | prevalence of burnout | **Expanders** - Apply related words; Apply equivalent subjects  **Search modes** - Proximity |
| S1 | prevalence of depression | **Expanders** - Apply related words; Apply equivalent subjects  **Search modes** – Proximity |

**Example of search in PsycINFO**

| **Search ID#** | **Search Terms** | **Search Options** |
| --- | --- | --- |
| S10 | S7 AND S8 | **Limiters** - Publication Date: 20210101-20241231  **Expanders** - Apply related words; Apply equivalent subjects  **Search modes** – Proximity |
| S9 | S7 AND S8 | **Expanders** - Apply related words; Apply equivalent subjects  **Search modes** - Proximity |
| S8 | S4 OR S5 OR S6 | **Expanders** - Apply related words; Apply equivalent subjects  **Search modes** - Proximity |
| S7 | S1 OR S2 OR S3 | **Expanders** - Apply related words; Apply equivalent subjects  **Search modes** - Proximity |
| S6 | resident physicians | **Expanders** - Apply related words; Apply equivalent subjects  **Search modes** - Proximity |
| S5 | medical practitioners | **Expanders** - Apply related words; Apply equivalent subjects  **Search modes** - Proximity |
| S4 | physicians or doctors or clinicians | **Expanders** - Apply related words; Apply equivalent subjects  **Search modes** - Proximity |
| S3 | prevalence of burnout | **Expanders** - Apply related words; Apply equivalent subjects  **Search modes** - Proximity |
| S2 | prevalence of anxiety | **Expanders** - Apply related words; Apply equivalent subjects  **Search modes** - Proximity |
| S1 | prevalence of depression | **Expanders** - Apply related words; Apply equivalent subjects  **Search modes** – Proximity |
